# Corrosion Behavior of Heat-Treated AlSi10Mg Manufactured by Laser Powder Bed Fusion

**DOI:** 10.3390/ma11071051

**Published:** 2018-06-21

**Authors:** Marina Cabrini, Flaviana Calignano, Paolo Fino, Sergio Lorenzi, Massimo Lorusso, Diego Manfredi, Cristian Testa, Tommaso Pastore

**Affiliations:** 1 Department of Engineering and Applied Sciences, University of Bergamo, 24044 Dalmine, Italy; sergio.lorenzi@unibg.it (S.L.); cristian.testa@unibg.it (C.T.); tommaso.pastore@unibg.it (T.P.); 2 INSTM Unità di Ricerca Bergamo, Dalmine, 24044, Italy; 3 Department of Management and Production Engineering, Politecnico di Torino, 10129 Torino, Italy; flaviana.calignano@polito.it; 4 Department of Applied Science and Technology, Politecnico di Torino, 10129 Torino, Italy; paolo.fino@polito.it; 5 Center for Sustainable Future Technologies CSFT@Polito, Istituto Italiano di Tecnologia (IIT), 10129 Torino, Italy; Massimo.Lorusso@iit.it (M.L.); diego.manfredi@iit.it (D.M.)

**Keywords:** additive manufacturing, Laser Powder Bed Fusion, intergranular corrosion, heat treatments, AlSi10Mg

## Abstract

This experimental work is aimed at studying the effect of microstructural modifications induced by post-processing heat treatments on the corrosion behavior of silicon-aluminum alloys produced by means of laser powder bed fusion (LPBF). The manufacturing technique leads to microstructures characterized by the presence of melt pools, which are quite different compared to casting alloys. In this study, the behavior of an AlSi10Mg alloy was evaluated by means of intergranular corrosion tests according to ISO 11846 standard on heat-treated samples ranging from 200 to 500 °C as well as on untreated samples. We found that temperatures above 200 °C reduced microhardness of the alloy, and different corrosion morphologies occurred due to the modification of both size and distribution of silicon precipitates. Selective penetrating attacks occurred at melt pool borders. The intergranular corrosion phenomena were less intense for as-produced specimens without heat treatments compared to the heat-treated specimens at 200 and 300 °C. General corrosion morphologies were noticed for specimens heat treated at temperatures exceeding 400 °C.

## 1. Introduction

High strength and stiffness, low weight, good formability, good corrosion resistance, and recycling possibility make aluminum alloys an ideal candidate to replace heavier materials (steel or copper) to satisfy the weight reduction demand in the automotive industry [1]. Morita reported that aluminum alloys exhibit an improvement in fuel economy of 5.5% for weight reduction of approximately 10% [2]. Recent examples of aluminum applications in vehicles cover power trains, chassis, body structure, and air conditioning. There are also strong predictions for aluminum applications in hoods, trunk lids, and steel frame doors [1,3].

In addition, there is now a general trend towards producing components in aluminum, titanium, and nickel alloys in additive manufacturing (AM) [4] owing to the relevant benefits of such techniques. This includes near-net-shape capabilities, superior design and geometrical flexibility, reduced tooling and fixturing, shorter cycle time for design and manufacturing as well as material, energy, and cost efficiency [5,6,7]. Atzeni and Salmi reported that AM is adequate for small to medium batch productions even for end-usable metal products. It reduces time and costs from the design phase to manufacturing because there is no investment in designing, production, and the necessary tools and fixtures [8].

Laser powder bed fusion (LPBF) is an AM technology for the fabrication of semi-finished components directly from computer-aided design modeling through melting and consolidation—layer upon layer—of a metallic powder with a laser source [9]. Recent studies have demonstrated that metallic AM parts/alloys experience a complex thermal history during production, which involves directional heat extraction, repeated melting and rapid solidification cycles, and repeated solid-state phase transformations. This introduces complexities to the analysis of microstructural evolution and properties not typically found in conventional processes [9].

Studies on development, properties, and microstructures of AM have mainly focused on titanium alloys—in particular Ti6Al4V [10] and nickel alloys [11]—owing to their high values, small production, and difficulties of machining. The aluminum alloy AlSi10Mg has also received attention as it can be conveniently obtained using LPBF.

AlSi10Mg alloy is a traditional cast alloy that is often used for die casting. Because of its near eutectic composition, it has good fluidity and low shrinkage. The magnesium in the alloy plays an important role in age-hardening, causing the formation of β’ phase and Mg_2_Si (β-phase) [12]. Various studies have been published on the microstructure of LPBF AlSi10Mg, starting from process parameters optimization [13,14,15].

In a previous study, Manfredi et al. [16] demonstrated that AlSi10Mg alloy specimens obtained by means of LPBF have higher hardness and yield strength compared to A360 cast alloy with similar composition. This is due to the very fine microstructure and fine distribution of silicon that is promoted by the extremely rapid cooling and solidification. This has also been reported by Thijs et al. [13]. Furthermore, Manfredi et al. [17] outlined a probable presence of Mg_2_Si intermetallic at nanometric scale. This was also noted by Olakanmi [18]. Read et al. [19] observed higher tensile strength of AlSi10Mg compared to A360 die cast in both building directions.

Moreover, the powder-based processes enable the consolidation of second or multiple material particles with metal powders to form metal matrix composites (MMCs) [20]. Particularly promising is AlSiMg alloy reinforced with SiC particles because the high amount of silicon reduces or suppresses the reaction between aluminum and silicon carbide. This is important as it prevents formation of the dangerous aluminum carbide Al_4_C_3_, which have a detrimental effect on the mechanical properties of the alloy [21].

The LPBF process leaves high residual thermal stresses in metal structures. In order to reduce them and avoid distortions of the manufactured object, stress relieving heat treatment at 300 °C for two hours is usually recommended [17]. However, negative effects on corrosion behavior have been reported. Previous studies conducted on stress-relieved specimens of AlSi10Mg [22,23,24,25] showed an increase in susceptibility to selective attack along the melt pool borders. Such morphology was not detected after annealing at 550 °C [23], which produces relevant variations of microstructure and reduces segregations in the critical thermally affected zone across the melt pools.

This paper reports the results of an experimental investigation aimed at studying the effect of heat treatments on microstructure and corrosion resistance of an AlSi10Mg alloy obtained by means of LPBF. The corrosion resistance was evaluated by means of intergranular corrosion tests according to ISO11846 standard. Microstructure and corrosion morphology were analyzed by means of optical and field emission scanning electron microscope (FESEM) equipped with energy dispersive X-ray spectroscopy (EDS).

## 2. Materials and Methods

The tests were carried out on samples obtained using a gas atomized AlSi10Mg powder produced by EOS Gmbh (Krailling, Germany). Table 1 reports the alloy composition. More detailed properties of the powders are described in previous works [16,17]. The particles have quite regular spherical shape ranging from 0.5 to 40 μm, with an average size of 25 μm.

During LPBF process, the laser beam intensity was adjusted to achieve complete melting of each powder layer and good connection between two consecutive layers. Furthermore, the scan strategy was optimized for reducing the porosity of samples [16], as stated by Aboulkhair et al. [26], Read et al. [19], and Louvis et al. [27]. The laser scanning direction was rotated by 67° between consecutive layers in order to achieve suitable overlapping and to ensure the highest density [17]. The scan speed, laser power, and hatching distance were optimized as described in a previous work [28].

**Figure 1:**
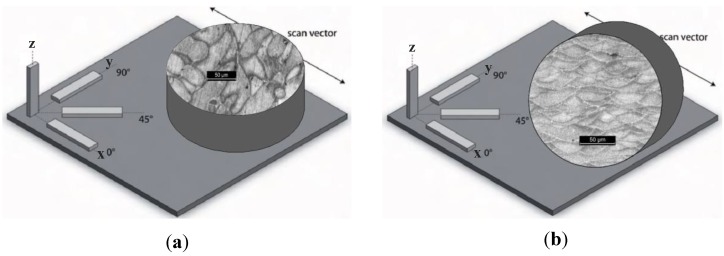
Schematic representation of specimens built with (**a**) the base parallel to building platform (XY specimens) and (**b**) perpendicular to the building platform (XZ specimens).

For the corrosion testing, cylindrical specimens with 15 mm diameter and 5 mm height were used. They were produced considering different orientations with respect to the building platform. Specimens were built both with the base parallel to the building platform (named XY, see Figure 1) and perpendicular to the building platform (named XZ, see Figure 1). Two different temperatures—35 °C and 100 °C—of the building platform during the production process were also considered to assess the role of this parameter on the corrosion behavior of the alloy.

The specimens without any further post-processing heat treatments were named UT (untreated). The heat treatments were carried out at 200, 300, 400, and 500 °C for 2 h, followed by cooling in still air.

Metallographic analysis was also carried out on specimens polished by means of abrasive papers up to 4000 grit, finished by 0.1 μm alumina aqueous suspension, and etched by means of Keller’s reagent.

Vickers microhardness tests were executed considering 100 g load and 15 s waiting time.

Intergranular corrosion tests were carried out according to ISO 11846 standard [29]. One side of the specimen was polished with emery paper up to 2400 grit, while the other side was left with the rough surface, i.e., as-produced by LPBF. As-built surfaces obtained by means of LPBF have high roughness, and it would have been very difficult to evaluate the depth of the selective attack. Previous papers [22,23,24] evidenced the difference in the corrosion behavior of the as-built and polished surface in the initiation of the corrosion attack owing to the differences in the passive film protectiveness. To avoid this issue, all specimens were pickled before testing and hence the original passive film did not influence the corrosion behavior. Then, the specimens were degreased with acetone in ultrasonic bath. According to the standard, the specimens were pickled in NaOH 8% at 55 °C for 3.5 min, rinsed in water, and dipped in 60% HNO_3_ for 2 min. They were then rinsed in distilled water and dried. All the reagents were of analytical grade.

The intergranular corrosion tests were carried in 30 g/L of NaCl solution with 10 mL/L of HCl at room temperature for 24 h. After exposure, the specimens were rinsed and the incoherent corrosion products were mechanically removed using a soft brush. They were then sectioned in order to analyze the morphology and depth of the attack.

## 3. Results and Discussion

Table 2 shows the results of our intergranular tests. The corrosion morphology is representative of the attacks that took place on all specimens. Preferential attacks at the melt pool borders can be evidenced in the first column of Table 2 (polished exposed surface). The depth of the attacks can be evidenced by the metallographic sections in the second and third columns of Table 2.

The specimens without any heat treatment did not show preferential attacks along the melt pools and the depth of penetration was negligible.

**Figure 2:**
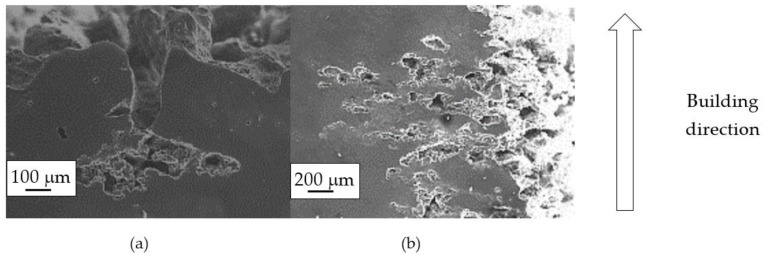
Metallographic section after ISO 11846 test on a specimen heat treated at 300 °C (platform 35 °C) built along XY plane (**a**) circular base of the specimens and (**b**) cylindrical lateral surface.

By contrast, the attack was deeper for specimens built at 35 °C compared to those built at 100 °C. The differences disappeared after the heat treatment. The samples heat treated at 200° C showed penetrating attack along the border of the melting pools, which became deeper and wider on the specimens heat treated at 300 °C. Such penetrating attacks were clearly evident on specimens built with the base perpendicular to the building platform (XZ), whereas the attack on the XY planes achieved lower depth because they deviated through the different layers (Figure 2). A previous paper [24] had indicated the noble pitting potential for XY specimens compared to XZ polished specimens heat treated at 300 °C. During our intergranular tests, the corrosion attack took place on all building directions due to the fact that the specimens were fully dipped in the testing solution. However, the exposure of both XY and XZ polished specimens allowed for better analysis of the morphology of the attack and enhanced the preferential growth of the attack with respect to the melted pool border. On the specimens heat treated above 400 °C, marked general corrosion attack to all the surfaces was observed independent of the building direction.

Preferential corrosion of the melt pool border can be attributed to the unique microstructure produced by LPBF process. Analysis of the corrosion morphologies was done at both macro and micro scales. The preferential attack of the melt pool borders could be clearly detected once the melt pool microstructure was determined by uneven silicon particle precipitation and element segregation. It became more penetrating when the heat treatment temperature increased from 200 to 300 °C. Such morphology disappeared after the temperature went above 400 °C.

**Figure 3:**
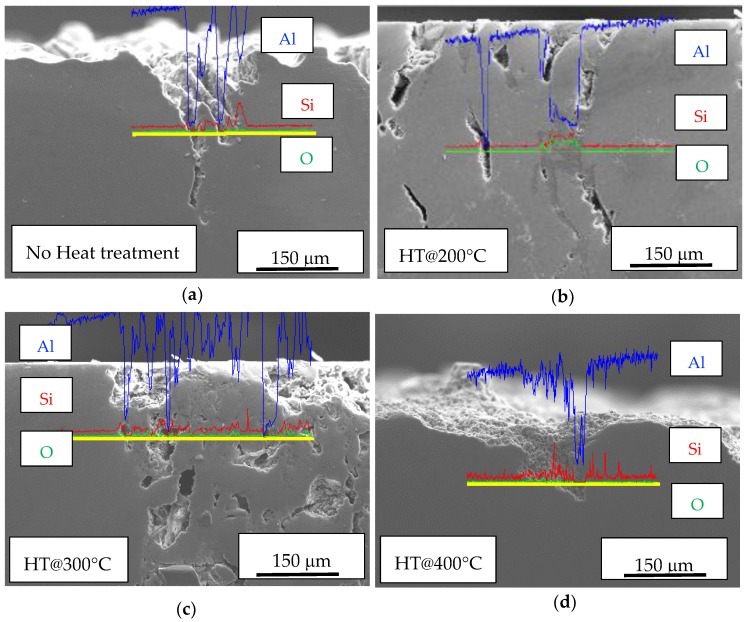
Energy dispersive X-ray spectroscopy (EDS) profile of aluminum (blue line), silicon (red line), and oxygen (green line) in the correspondence of attacks on specimens (**a**) not heat treated; (**b**) treated at 200 °C for 2 h; (**c**) treated at 300 °C for 2 h; (**d**) treated at 400 °C for 2 h.

The EDS profiles of Al, Si, and O showed the depletion of aluminum and the enrichment of silicon in the correspondence of the attack (Figure 3). The selective dissolution of α-aluminum phase with respect to silicon particles is due to the higher practical nobility of the silicon particles [30].

The galvanic effect of silicon particles on aluminum has been previously demonstrated by means of Scanning Kelvin Probe Microscopy [31]. Osorio et al. [32] emphasized that the increase in silicon concentration of hypoeutectic Al/Si alloys leads to an increase in the corrosion rate as the fraction of eutectic component increases.

### 3.1. Macrostructure

**Figure 4:**
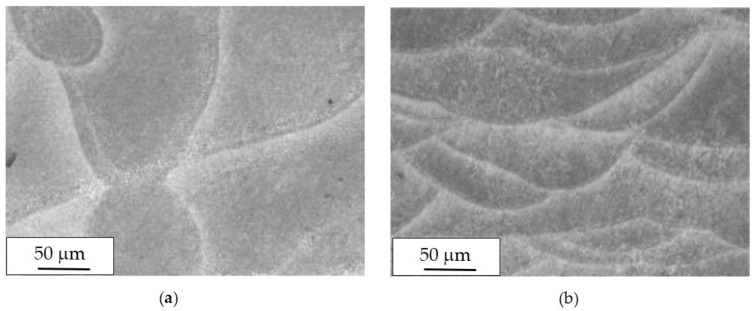
Optical images of microstructures of untreated (UT) specimen on (**a**) XY plane and (**b**) XZ plane.

The macrostructure of the UT samples along the building plane (XY) showed the tracks of the laser scan. The melt pool borders were elongated due to the variation of the laser scan direction—which rotates by 67° between consecutive layers—during building (Figure 4a). The temperature of the platform did not modify the alloy macrostructure.

Along the Z-direction, the macrostructure showed semicircular shaped melt pools, partially overlapped on previous tracks (Figure 4b).

During the LPBF process, the underlying metal, which was only superficially melted, quickly removed the heat from the liquid and ensured the adhesion of the track to the substrate. Each singular track could be considered as a small casting deposited on the previous one.

**Figure 5:**
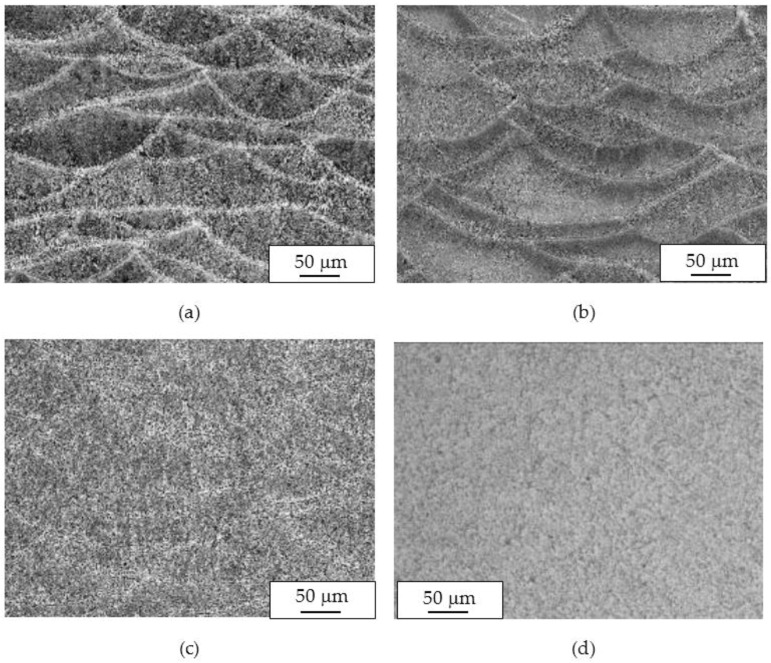
Macrostructure of XZ plane specimens after 2 h of heat treatment at different temperature: (**a**) 200 °C; (**b**) 300 °C; (**c**) 400 °C; and (**d**) 500 °C.

The macrostructures of the specimens did not change after heat treatments at temperatures of 200 and 300 °C, and the melt pools were still visible (Figure 5). When the heat treatment temperature was increased to 400 °C, the melt pool borders could not be well distinguished and practically disappeared at 500 °C. After heat treatment at this temperature, the macrostructure was significantly modified, and the α-Al phase matrix with rounded particles of silicon could be noticed. The size of these particles increased with time and heat treatment temperatures. At the highest temperature, second phase separations inside of the α-Al phase could be detected.

The microstructure of the AlSi10Mg obtained by LPBF has been described in previous work by other authors [33]. The microstructure modification inside the melt pools depends on both the rapid solidification process and directional cooling. SEM analysis revealed cellular grains inside the melt pools and oriented dendrites.

**Figure 6:**
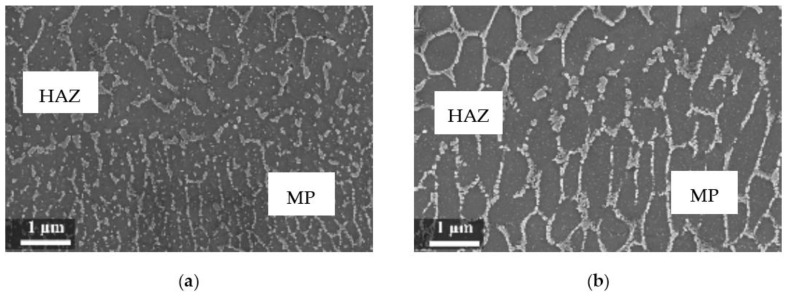
Microstructure of as-built specimens using different temperatures of the building platform (**a**) 35 °C and (**b**) 100 °C.

The rapid solidification inhibited segregation and precipitation of alloying elements from liquid and solid solution and produced a microstructure far from equilibrium. The directional cooling affected the grain growth perpendicularly with respect to the melt pool border towards the center of the melt pool [9]. Smaller equiaxed grains were observed at the center of the melt pool, while the border of the melt pool—where two tracks were overlapped—underwent double melting. Heat Affected Zone (HAZ) could be noticed just next to the melt pool border (Figure 6). This has also been described by Thijs et al. [13].

### 3.2. Microstructure

The silicon distribution in the melt pool is regulated by the heating and cooling rate of the alloy. Owing to the high solidification rate, the α-Al phase inside the melt pool remains oversaturated in silicon [34,35]. According to Murray and McAlister, the maximum solubility of silicon in aluminum is about 1.5 wt % at the eutectic temperature but decreases to less than 0.05 wt %. below 300 °C. Rapid cooling can maintain the silicon content into an α-Al phase at oversaturated level up to 11 wt % [36].

**Figure 7:**
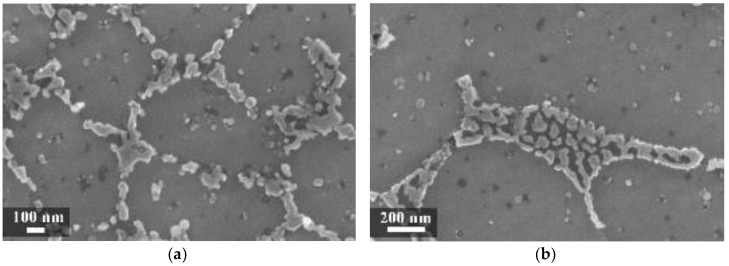
Close-up of the melt pool (MP) zones (**a**) of the silicon-isolated particles in HAZ; (**b**) of the as-built specimens (T_platform_ 35 °C).

**Figure 8:**
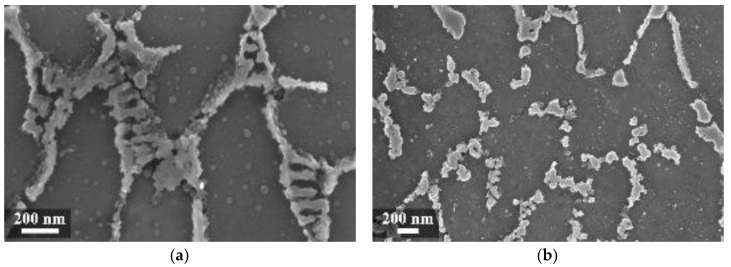
(**a**) Close-up of the eutectic-like phase on the border of α-Al grains and (**b**) idiomorphic crystals in the HAZ of the as-built specimens (T_platform_ 100 °C).

In our experiment, during LPBF the silicon did not fully remain in solid solution, but partially segregated on the columnar grains of the α-Al phase as fibrous particles that “decorate” the primary grains of the α-Al phase (Figure 7a) [35]. Thijs et al. defined the structure of silicon as “intercellular network” [13]. At times, the silicon particles formed a eutctic-like phase (Figure 7). The eutectic was more visible in specimens built with a platform temperature of 100 °C compared to 35 °C (Figure 7 and Figure 8).

**Figure 9:**
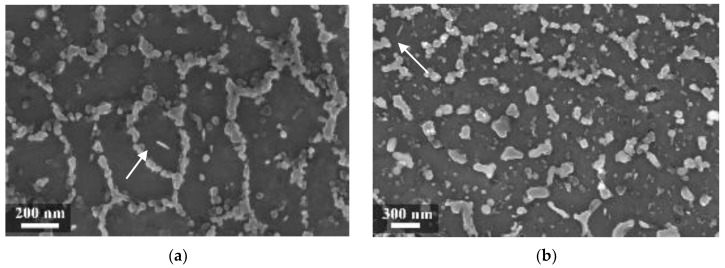
Microstructure of the AlSi10Mg specimen after stress relieved at 300 °C (**a**) MP and (**b**) HAZ (T_platform_ 35 °C).

In the HAZ, the solid state diffusion was promoted by thermal cycles and interruptions in the intercellular network occurred. Silicon particles coarsening could be noticed, which became idiomorphic crystals (Figure 9b) [13].

The heat treatments at 200 and 300 °C did not modify the macrostructure but favored silicon precipitation. An increase in the silicon particle density occurred, but their size remained noticeably small. Some isolated particles became visible inside the α-phase grain.

The heat treatments completely erased the slight differences between the microstructure of specimens built by considering different temperatures of the building platform.

The preferential attack at the melt pool border was already observed on heat-treated specimens at 300 °C for 2 h [22,23] and without heat treatment [37]. Cabrini et al. [22] stated that the enhanced content of silicon in the solid solution in the melt pool (due to the rapid solidification and cooling) increases corrosion resistance and the formation of preferential dissolution path in HAZ due to the separation of silicon idiomorphic crystals and produces local galvanic couples. The silicon network that decorates the aluminum dendrites inside the melt pool could partially hinder the aluminum matrix by decelerating the corrosion rate. Conversely, isolated particles in the HAZ stimulate α-Al corrosion. For this reason, the dissolution rate of the melt pool border was higher than the center. The heat treatments at 200 and 300 °C promoted an increase in size of silicon crystals around the α-Al phase at the border of the melt pool, thus enhancing the intensity of the attacks.

Revilla et al. [37] observed a close relationship between the size of the cellular grains and the Volta potential difference between the silicon and the aluminum phase. Greater potential difference between the phases was found in the regions with larger and coarser microstructures, which represent a higher driving force for galvanic corrosion. This could explain why corrosion attacks arise in the melt pool borders where larger microstructures are generally found.

**Figure 10:**
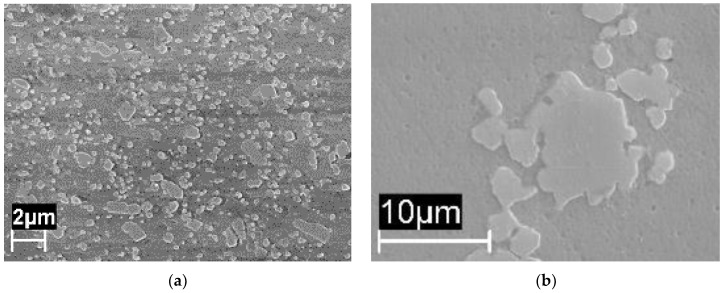
Microstructure of the XZ (T_platform_ 35 °C) specimens heat treated at (**a**) 400 °C and (**b**) 550 °C [38].

In our experiment, when the heat treatment temperature was further increased to 400 °C (Figure 10a) and 500 °C, the silicon particle coalescence occurred and rounded second phases were formed.

Hence, the galvanic effect of silicon particles became prevalent over the mechanisms occurring at the melt pool border. Corrosion attack was localized at the interface between the silicon precipitates and aluminum matrix. The even distribution of precipitates promoted intense general corrosion attack, with shallow and broad pits at most.

Annealing heat treatment at 550 °C completely destroyed the melt pool structure [23] and gave rise to α-Al phase matrix with coarse round-shaped silicon precipitates inside with sizes ranging from 1 to 10 μm. This has been noted by Ogris et al. [38].

### 3.3. Effect of Second Phases

In literature, the effect of magnesium and iron precipitates on the aluminum corrosion is well known [39,40,41] even though both elements are present in this alloy in low quantities. In regards to the role of these elements in the corrosion behavior, it was observed that Mg_2_Si precipitates were never detected in the analysis by SEM independently of the heat treatment. However, Manfredi et al. [17] remarked that iron and magnesium precipitates should be so fine that it is impossible to detect them by either metallographic attack or EDS analysis.

The alloy used for this experimental work contained approximately 0.16% iron by weight. On the as-built specimens, there was no evidence of iron phases. However, some needle-like microparticles could be detected in the center of the α-Al phase grains after stress relieving at 300 °C, mainly in the coarse oriented grain zone (arrows in Figure 9). Their morphology can be related to iron intermetallic phases, as reported by Takata et al. [34].

In Al-Si based alloys, the size of the β-Al5FeSi intermetallic phase is directly dependent on the iron content and cooling rate. The size of β-iron intermetallic phases increases with the iron content and decreases with cooling rate [42]. Their presence in the coarse oriented grain zone can be ascribed to the lower solidification rate. Unfortunately, the grain size and particle dimensions are too small, so it is impossible to detect differences in their composition using EDS. Holesinger et al. [43] stated that iron and magnesium concentrations were higher at the border of the melt pool, with iron preferentially located at α-aluminum grain boundary. Shankar et al. [44] have said that iron promotes the formation of the α-Al/Si eutectic.

Silicon particles are not an efficient cathode for oxygen and hydrogen reduction reactions, but iron particles can be very efficient in promoting the preferential dissolution of aluminum [45].

On the specimens heat treated at 300 °C, needle-like phases were detected after further annealing at 550 °C. The size of these phases was large enough to permit EDS analysis, which confirmed the presence of iron.

### 3.4. Microhardness

**Figure 11:**
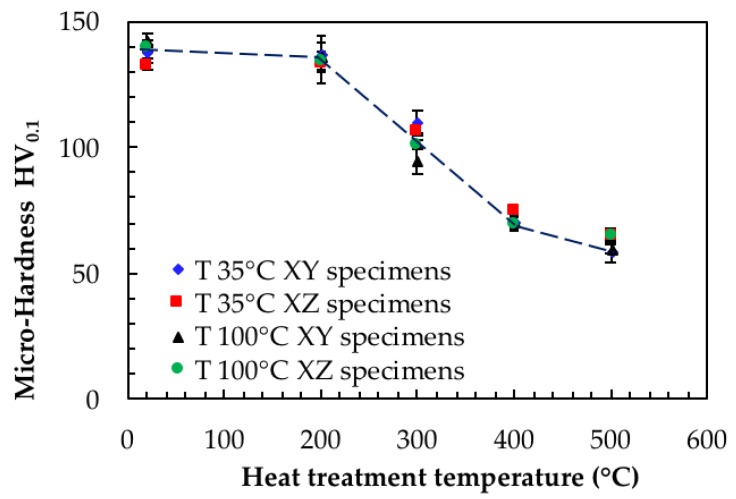
Effect of thermal treatments on microhardness.

The role of heat treatment was very important for both the corrosion behavior and the mechanical properties of the alloy. Very fine microstructure obtained by means of LPBF process led to high hardness values, but the heat treatments significantly affected microstructure (Table 3 and Figure 11). Our data confirmed the decrease in the mechanical properties as the temperature of the heat treatment increased. Furthermore, they did not show any relevant differences between XY and XZ planes and between the specimens built using different platform temperatures. This finding is of particular importance because it must be taken into account that materials properties—both mechanical and corrosion performance—have to be considered while developing suitable heat treatments for optimization of specific applications.

Stress relieving at 200 °C for 2 h only slightly affected hardness, but heat treatments at temperature of 300 °C, which are normally considered for stress relieving, significantly affected the hardness values up to about 25–30% compared to the untreated alloy.

Takata et al. reported a significant reduction in the strength of the as-fabricated specimen after heat treatment at 300 °C for 2 h, regardless of the slight differences in the microstructure as detected by SEM and electron backscatter diffraction (EBSD) analyses [34]. They suggested two possible reasons for the reduced strength: (1) the destruction of the fine sub-structures of a tangled array of dislocations within the α-Al elongated grains; and (2) the precipitation of the fine Si phase during annealing.

Differential scanning calorimetry (DSC) analysis carried out by Marola et al. [46] provided evidence of precipitation of excess Si solutes in temperatures ranging from ~150 to 350 °C. Further exothermic signals were attributed to the precipitation of Mg_2_Si (signal detected around 317 °C) and possibly Fe-containing intermetallics around 340 °C. The precipitation of these second phases should increase the hardness but the major reduction of silicon oversaturation of aluminum matrix counteracts this effect.

In our study, a further increase of heat treatment temperatures decreased the hardness of the alloy owing to the separation of the silicon phase.

The results emphasize that the best mechanical and corrosion performances can be evidenced on the as-built alloy. Increasing the building platform from 35 to 100 °C can reduce internal residual stresses and does not affect the mechanical strength or susceptibility to selective corrosion attack. Therefore, the use of high building platform temperature—i.e., up to 200 °C—is advisable in order to avoid stress relieving heat treatments [47].

## 4. Conclusions

In this study, the effect of heat treatment on the microstructure and the corrosion resistance of AlSi10Mg alloy produced by means of LPBF was evaluated through selective corrosion tests. The results emphasized the better mechanical and corrosion behavior of the as-built alloy.

Susceptibility of alloy to selective corrosion attack was noticed after stress relieving in the range of 200–300 °C—temperatures that are commonly recommended for post-processing heat treatments such as stress relieving. The attack occurred through the dissolution of α-Al phase stimulated by the precipitation of cathodic silicon particles, with preferential attack on the melt pool borders. The attack intensity achieved a maximum after heat treatment at 300° C.

High temperature heat treatments at 400° C and 500 °C produced marked decrease in hardness, but they prevented the insurgence of selective corrosion attack. General corrosion morphologies were noticed. Heat treatments at 200 and 300 °C for 2 h maintained the characteristic melt pool macrostructure, but these disappeared above 400 °C. At higher temperature, the microstructure revealed α-Al matrix with silicon rounded particles; the size increased with the temperature of heat treatment.

The study has found that increasing the building platform from 35 to 100 °C reduces internal residual stresses and does not affect mechanical strength or susceptibility to selective corrosion attack. Therefore, the use of high building platform temperature—i.e., up to 200 °C—is advisable in order to avoid stress relieving heat treatments.

## Figures and Tables

**Table 1:** Chemical composition (% weight) of the alloy.

Chemical Composition	Content (% Weight)
Si	10.1
Fe	0.16
Cu	0.001
Mn	0.002
Mg	0.35
Zn	0.002
Ti	0.01

**Table 2:** Morphology of corrosion after ISO 11846 test.

Heat Treatment	Exposed Surface (T 35 °C) (xz plane)	Metallographic Section (Exposed Surface xy, Section xz Plane)	
		Platform Temperature 35 °C	Platform Temperature 100 °C
No	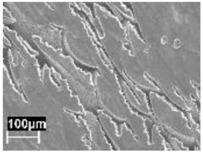	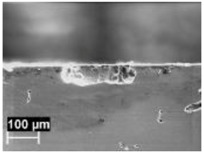	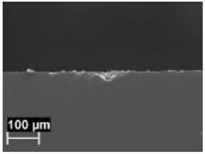
200 °C	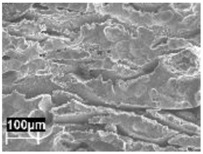	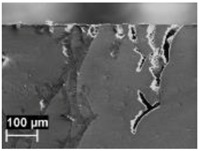	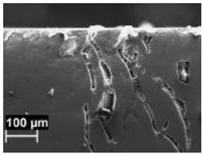
300 °C	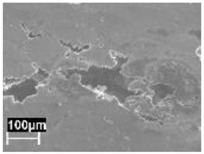	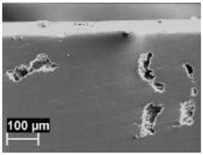	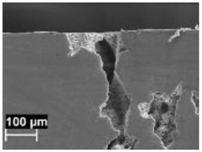
400 °C	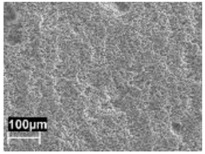	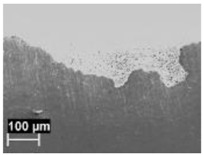	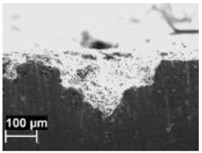
500 °C	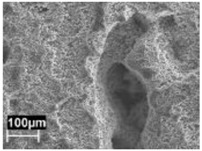	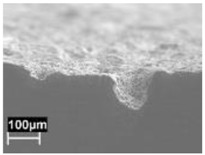	

**Table 3:** Results of microhardness measurements (HV_0.1_).

Heat Treatment		Cooling	Temperature of Building Platform			
			100 °C		35 °C	
T (°C)	t (h)		XZ	XY	XZ	XY
UT	-	-	143 ± 3	140 ± 2	138 ± 2	132 ± 1
200	2	air	136 ± 5	135 ± 4	137 ± 7	134 ± 8
300	2	air	95 ± 5	101 ± 2	110 ± 4	106 ± 1
400	2	air	70 ± 2	69 ± 3	71 ± 2	75 ± 3
500	2	air	60 ± 2	65 ± 3	59 ± 4	65 ± 2
